# Antioxidative and Anti-Atopic Dermatitis Effects of Peptides Derived from Hydrolyzed *Sebastes schlegelii* Tail By-Products

**DOI:** 10.3390/md22100479

**Published:** 2024-10-19

**Authors:** Sung-Gyu Lee, Jin-Woo Hwang, Hyun Kang

**Affiliations:** 1 Department of Medical Laboratory Science, College of Health Science, Dankook University, Cheonan-si 31116, Chungcheongnam-do, Republic of Korea; croucard@naver.com; 2 Marine Bio-Food and Drug Convergence Technology Center, Dankook University, Cheonan-si 31116, Chungcheongnam-do, Republic of Korea

**Keywords:** *Sebastes schlegelii* tail by-products, atopic dermatitis, peptides, STAT3, antioxidant

## Abstract

Atopic dermatitis (AD) is a chronic inflammatory skin disorder associated with significant morbidity, including pruritus, recurrent skin lesions, and immune dysregulation. This study aimed to investigate the antioxidative and anti-AD effects of peptides derived from hydrolyzed *Sebastes schlegelii* (Korea rockfish) tail by-products. Hydrolysates were prepared using various enzymes, including Alcalase, Flavourzyme, Neutrase, and Protamex. Among them, Protamex hydrolysates demonstrated the highest ABTS radical scavenging activity with an RC_50_ value of 69.69 ± 0.41 µg/mL. Peptides were further isolated from the Protamex hydrolysate using dialysis, fast protein liquid chromatography (FPLC), and high-performance liquid chromatography (HPLC). The most active peptide, STPO-B-II, exhibited a single peak and was identified as a sequence of Glu-Leu-Ala-Lys-Thr-Trp-His-Asp-Met-Lys, designated as MP003. In vivo experiments were conducted using a 2,4-dinitrochlorbenzene (DNCB)-induced AD model in NC/Nga mice. The isolated peptide, MP003, showed significantly reduced AD symptoms, including erythema, lichenification, and collagen deposition. Additionally, MP003 decreased epidermal and dermal thickness, eosinophil, and mast cell infiltration and downregulated the expression of pro-inflammatory cytokines IL-1β, IL-6, and IgE in serum and skin tissues. These findings suggest that peptides derived from *Sebastes schlegelii* tail by-products may serve as potential therapeutic agents for AD.

## 1. Introduction

Atopic dermatitis (AD) is a chronic inflammatory skin disease characterized by persistent pruritus and recurrent skin lesions, affecting up to 20% of children and 10% of adults in high-income countries [1,2]. Impaired skin barrier function and immune system dysregulation are key contributors to AD pathogenesis [3]. The intermittent and chronic disease course, severe pruritus, rash, and complications such as depression, anxiety, and sleep disturbances can greatly affect the quality of life [4]. Moreover, AD is associated with multiple comorbidities, including allergies, infections, and cardiovascular complications [5]. Although monoclonal antibodies, topical agents, and small-molecule inhibitors have been introduced for AD treatment in recent years [6], there remains a need for novel therapeutic agents to enhance disease control.

Global aquaculture and fisheries production in 2018 reached 178.5 million tons, with 87.6% consumed by humans [7]. This figure is projected to rise annually due to population growth and increasing awareness of marine health benefits. However, the fish processing industry generates substantial by-products, including heads, tails, skin, scales, viscera, and bones [8,9]. It is estimated that 35% of harvested fish are lost during processing, with 70% becoming by-products [7]. Globally, efforts are underway to recycle these by-products in an environmentally sustainable manner [10,11].

Fish by-products have also been studied for their potential in medical and therapeutic applications due to the bioactive compounds they contain, including peptides, collagen, and fatty acids [7,12,13]. For instance, collagen derived from fish is known for its hypoallergenic properties, which makes it suitable for use in sensitive skin conditions like AD [14,15]. Fish oils, rich in omega-3 fatty acids, have also shown potential in alleviating AD symptoms by reducing skin inflammation and improving barrier function [16]. Additionally, protein hydrolysates from fish by-products are often rich in bioactive peptides, which have antioxidative and anti-inflammatory properties [17,18,19]. Protein hydrolysates typically contain low-molecular-weight peptides composed of 2–20 amino acids [20], and their bioactivity varies depending on the amino acid composition and sequence of the source material [21]. Marine organisms living in complex habitats and exposed to extreme conditions produce bioactive peptides with unique amino acid sequences compared to terrestrial organisms [22,23,24].

*Sebastes schlegelii*, commonly known as the black rockfish, is a commercially valuable species found in the Yellow Sea, East China Sea, the Korean Peninsula, and the coasts of Japan [25,26]. In aquaculture, body parts such as the head, tail, and bones are underutilized. Repurposing these by-products could minimize waste, reduce environmental pollution, and enhance economic benefits [10,11].

This study aimed to utilize the tail by-products of *Sebastes schlegelii* to produce protein hydrolysates through enzymatic hydrolysis, isolate antioxidant peptides, and evaluate their potential as a novel therapeutic material for atopic dermatitis using a 2,4-dinitrochlorbenzene (DNCB)-induced AD mouse model.

## 2. Results

### 2.1. Yield and ABTS Radical Scavenging Activity of Hydrolysates from Sebastes schlegelii Tail

The yield and 2,2′-azino-bis(3-ethylbenzothiazoline-6-sulfonic acid) (ABTS) radical scavenging activity results of *Sebastes schlegelii* tail hydrolysates are shown in Table 1. The yields of hydrolysates using Alcalase, Flavourzyme, Neutrase, and Protamex were 64.41%, 75.21%, 73.36%, and 72.09%, respectively, with the highest yield obtained using Flavourzyme. The ABTS radical scavenging activity of the *Sebastes schlegelii* tail hydrolysates, expressed as RC_50_ values (the concentration required to inhibit 50% of radicals), was 79.64 ± 2.64, 77.23 ± 2.17, 97.70 ± 4.46, and 69.69 ± 0.41 for Alcalase, Flavourzyme, Neutrase, and Protamex, respectively. The hydrolysates using Protamex showed the most effective ABTS radical scavenging activity.

### 2.2. Isolation of Antioxidant Peptides from Sebastes schlegelii Tail Hydrolysates

**Figure 1:**
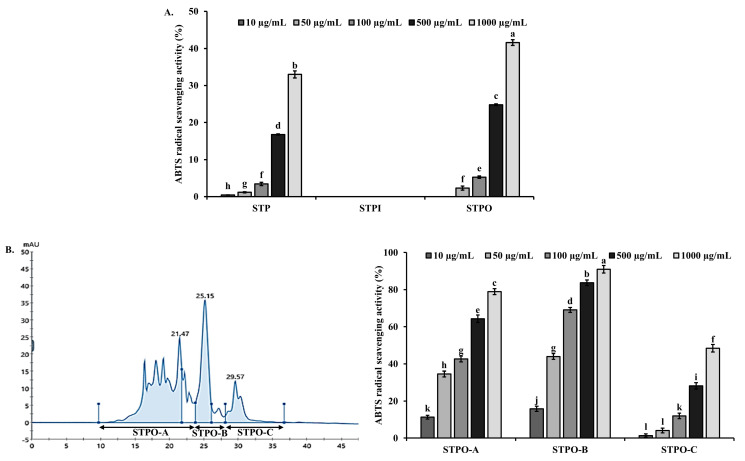

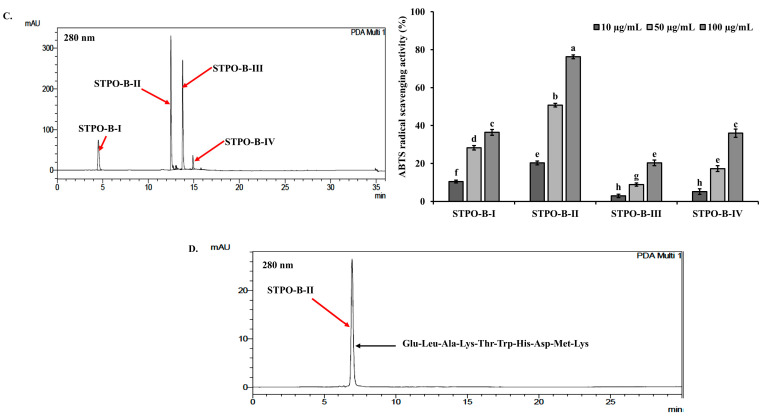
Purification of Protamex hydrolysate (STP). (**A**) ABTS radical scavenging activities of hydrolysate both inside (STPI, ≥3500 Da) and outside (STPO, <3500 Da) the 3500 Da dialysis membrane. (**B**) The FPLC pattern on the GPC column of the STPO active fraction and the ABTS radical scavenging activities of the fractions. (**C**) The reversed-phase HPLC pattern of the STPO-B active fraction and the ABTS radical scavenging activities of the fractions. (**D**) The HPLC pattern with a GPC column of the STPO-B-II active fraction. Each value is the mean ± SD (*n* ≥ 3). Different superscripts (a–l) in the same column are significantly different at *p* < 0.05, using Duncan’s multiple range test.

Peptide isolation was performed using *Sebastes schlegelii* tail hydrolysates prepared with Protamex, which showed the highest ABTS radical scavenging activity. In the first step, the hydrolysates were separated using a 3500 Da dialysis membrane into inner (STPI: *Sebastes schlegelii* Tail Protamex hydrolysate inner membrane, ≥3500 Da) and outer (STPO: *Sebastes schlegelii* Tail Protamex hydrolysate outer membrane, <3500 Da) fractions. The ABTS radical inhibition activity assay showed that the STPO fraction exhibited a higher ABTS radical inhibition than the original Protamex hydrolysate (STP) (Figure 1A). The STPO fraction, which showed enhanced ABTS radical scavenging activity, was further separated using fast protein liquid chromatography (FPLC) equipped with a GPC column. Three fractions (STPO-A, STPO-B, STPO-C) were collected, and their ABTS radical scavenging activities were measured (Figure 1B). Among these, STPO-B showed the highest ABTS radical scavenging activity, and this fraction was subjected to further separation. STPO-B was fractionated using high-performance liquid chromatography (HPLC) with a C18 column, yielding four fractions: STPO-B-I, STPO-B-II, STPO-B-III, and STPO-B-IV. The ABTS inhibition activity of these four fractions was measured, with STPO-B-II exhibiting the highest ABTS radical scavenging activity (Figure 1C). The further separation of STPO-B-II using HPLC with a GPC column resulted in a single purified fraction (Figure 1D). The amino acid sequence of the purified peptide, identified using a Milligen 6600 protein sequencer, was Glu-Leu-Ala-Lys-Thr-Trp-His-Asp-Met-Lys. This peptide was designated MP003 (Marine Peptide 003). The identified peptide was synthesized for animal experiments by A&Pep Co., Ltd. (Cheongju-si, Chungcheongbuk-do, Republic of Korea).

### 2.3. Effects of MP003 on Symptoms of AD in DNCB-Induced NC/Nga Mice

**Figure 2:**
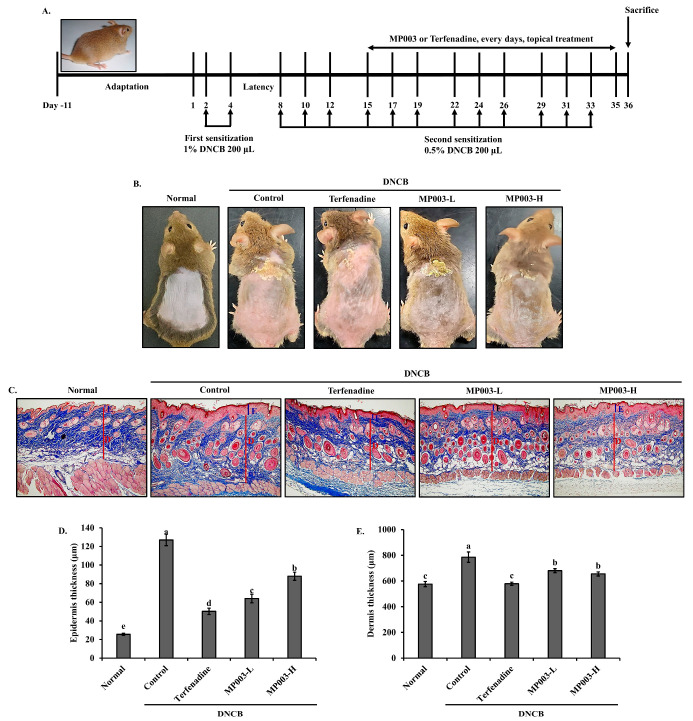
The effects of MP003 on the histological characteristics of AD in DNCB-induced NC/Nga mice. (**A**) A schematic diagram of the experimental schedule. (**B**) Clinical characteristics of each treatment group on day 36. (**C**) Epidermal (**E**) and dermal (**D**) thickness was examined by Masson’s trichrome staining of skin sections. Magnification, ×100. The measurement of (**D**) epidermal and (**E**) dermal thickness. Each value is the mean ± SD (*n* = 5 per group). Different superscripts (a–e) in the same column are significantly different at *p* < 0.05, using Duncan’s multiple range test. Normal, untreated group; Control, DNCB-sensitized group; Terfeadine, 250 μM Terfenadine-treated group; MP003-L, 125 μM MP003-treated group; and MP003-H, 250 μM MP003-treated group.

We investigated the therapeutic effects of MP003 in a DNCB-induced AD model using the dorsal skin of NC/Nga mice (Figure 2). All animal experiments were approved by the Dankook University Institutional Animal Care and Use Committee (IRB No: DKU-23–023). Six-week-old male NC/Nga mice were purchased from ORIENT (Seongnam, Republic of Korea). Mice were acclimatized for 9 days under controlled temperature (23 ± 2 °C) and humidity (50 ± 10%). Mice were divided into five groups: Normal, untreated group; Control, DNCB-sensitized group; Terfenadine, 250 μM Terfenadine-treated group; MP003-L, 125 μM MP003-treated group; and MP003-H, 250 μM MP003-treated group. AD was induced using 0.5% and 1% DNCB diluted in a solvent of acetone and olive oil (3:1). After shaving the backs of the mice, 200 μL of 1% DNCB was applied on days 2 and 4, followed by a sensitization phase for four weeks. Mice in the non-atopic group received a solvent of olive oil and PBS (1:9) (Figure 2A). Typical AD symptoms such as erythema and lichenification were significantly increased in the DNCB-induced control group. By contrast, both the MP003-L and MP003-H groups exhibited a reduction in the severity of erythema and lichenification throughout the study period (Figure 2A). Collagen accumulation in the NC/Nga mouse model of AD is crucial, as it directly reflects the extent of skin fibrosis in AD-like lesions. Masson’s trichrome staining results (Figure 2B) revealed excessive collagen deposition and fibrosis (stained blue) in the dermis of the DNCB-induced control group. However, collagen fiber accumulation was reduced in both the Terfenadine and MP003-treated groups, indicating that MP003 treatment effectively alleviates skin fibrosis. In addition, the changes in epidermal and dermal thickness were assessed. The DNCB-induced control group exhibited a significant (*p* < 0.05) increase in both epidermal and dermal thickness compared to the Normal group. Conversely, the MP003-treated groups showed a significant (*p* < 0.05) reduction in epidermal and dermal thickness compared to the control group (Figure 2C,D).

### 2.4. Effect of MP003 on Eosinophil and Mast Cell Infiltration in Skin Tissues of DNCB-Induced NC/Nga Mice

**Figure 3:**
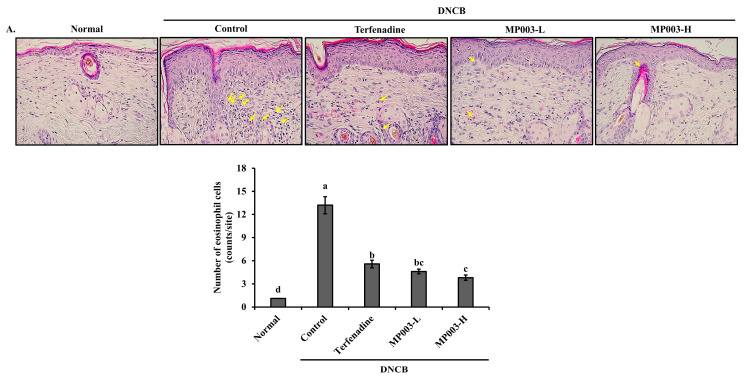

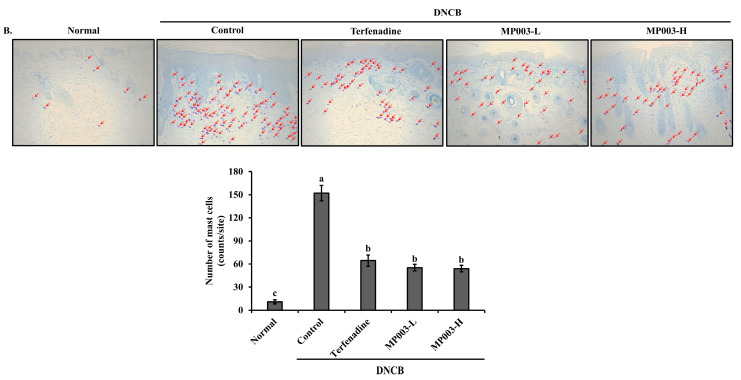
The effects of MP003 on the infiltration and numbers of eosinophils and mast cells in DNCB-induced NC/Nga mice on day 36. Infiltration by (**A**) eosinophils (yellow arrow; magnification, ×400) and (**B**) mast cells (red arrow; magnification, ×200) in dermal lesions were examined by the H&E and toluidine blue staining of skin sections. Each value is the mean ± SD (*n* = 5 per group). Different superscripts (a–d) in the same column are significantly different at *p* < 0.05 by Duncan’s multiple range test. Normal, untreated group; Control, DNCB-sensitized group; Terfeadine, 250 μM Terfenadine-treated group; MP003-L, 125 μM MP003-treated group; and MP003-H, 250 μM MP003-treated group.

To assess the effect of MP003 on DNCB-induced eosinophil and mast cell infiltration into the skin lesions of NC/Nga mice, dorsal skin sections collected on the final day of the experiment (day 36) were stained with Hematoxylin and Eosin (H&E) and toluidine blue to identify eosinophils and mast cells, respectively. The number of eosinophils stained with H&E was significantly (*p* < 0.05) increased in the skin lesions of the DNCB-induced control group compared to the Normal group (Figure 3A). In contrast, the topical application of MP003, as well as Terfenadine, significantly (*p* < 0.05) reduced the number of eosinophils in the dorsal skin of DNCB-treated NC/Nga mice (Figure 3A). Mast cell infiltration, a marker of inflammation, was observed in toluidine blue-stained skin sections. The number of mast cells was significantly (*p* < 0.05) increased in the DNCB-induced control group compared to the Normal group, while MP003 treatment resulted in a reduction in mast cell numbers (Figure 3B). These results suggest that topically applied MP003 has the potential to improve the histological characteristics of the skin in DNCB-induced AD mice.

### 2.5. Effect of MP003 on Cytokine and IgE Levels in the Serum of DNCB-Induced NC/Nga Mice

**Figure 4:**
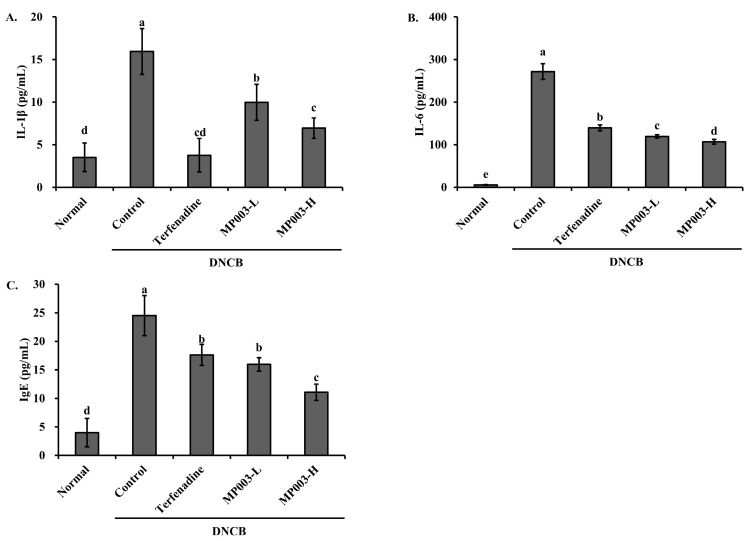
The effects of MP003 on serum (**A**) IL-1β, (**B**) IL-6, and (**C**) IgE levels in DNCB-induced NC/Nga mice on day 36. Each value is the mean ± SD (*n* = 5 per group). Different superscripts (a–d) in the same column are significantly different at *p* < 0.05, using Duncan’s multiple range test. Normal, untreated group; Control, DNCB-sensitized group; Terfeadine, 250 μM Terfenadine-treated group; MP003-L, 125 μM MP003-treated group; and MP003-H, 250 μM MP003-treated group.

To investigate the effect of MP003 on serum cytokine and Immunoglobulin E (IgE) levels, the concentrations of Interleukin-1 beta (IL-1β), IL-6, and IgE were measured (Figure 4). The DNCB-induced control group exhibited significantly elevated levels of IL-1β, IL-6, and IgE in the serum compared to the Normal group (*p* < 0.05). However, treatment with Terfenadine and MP003 significantly (*p* < 0.05) reduced the levels of IL-1β, IL-6, and IgE in all experimental groups. These results suggest that MP003 effectively reduces cytokine and IgE production in DNCB-induced AD mice.

### 2.6. Effect of MP003 on IL-1β and IL-6 Gene Expression in the Skin Tissues of DNCB-Induced NC/Nga Mice

**Figure 5:**
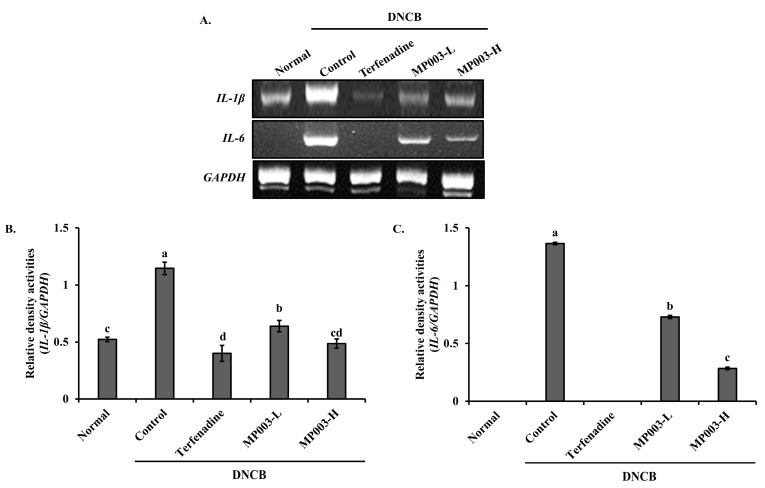
The effects of MP003 on the gene expression of *IL-1β* and *IL-6* in the dorsal skin in DNCB-induced NC/Nga mice on day 36. (**A**) Representative RT-PCR bands for *IL-1β, IL-6,* and *GAPDH* in the dorsal skin. (**B**,**C**) quantitative analysis of RT-PCR bands. Each value is the mean ± SD (*n* = 5 per group). Different superscripts (a–d) in the same column are significantly different at *p* < 0.05, using Duncan’s multiple range test. Normal, untreated group; Control, DNCB-sensitized group; Terfeadine, 250 μM Terfenadine-treated group; MP003-L, 125 μM MP003-treated group; and MP003-H, 250 μM MP003-treated group.

The changes in the expression of inflammation-related genes *IL-1β* and *IL-6* in the skin tissues of DNCB-induced AD mice were measured using the reverse transcription–polymerase chain reaction (RT-PCR) (Figure 5). In the DNCB-induced control group, the expression levels of *IL-1β* and *IL-6* were significantly (*p* < 0.05) elevated compared to the Normal group. However, treatment with Terfenadine and MP003 significantly (*p* < 0.05) decreased the expression levels of *IL-1β* and *IL-6* in the experimental groups. These results indicate that MP003 effectively suppresses the gene expression of pro-inflammatory cytokines in the skin tissues of DNCB-induced AD mice.

### 2.7. Effect of MP003 on STAT3 Protein Expression in the Skin Tissues of DNCB-Induced NC/Nga Mice

**Figure 6:**
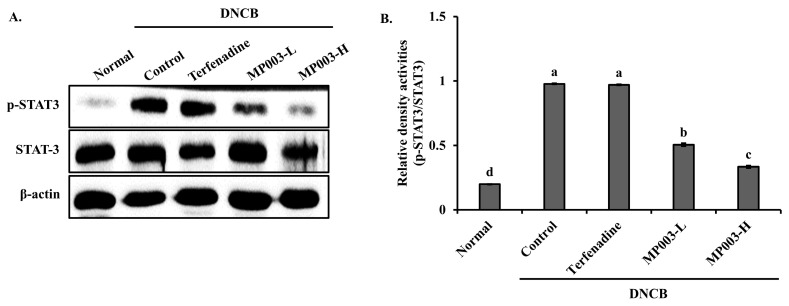
The effects of MP003 on the protein expression of STAT3 in the dorsal skin in DNCB-induced NC/Nga mice on day 36. (**A**) Representative Western blot bands for p-STAT3, STAT3, and β-actin in the dorsal skin. (**B**) Quantitative analysis of Western blot bands. Each value is the mean ± SD (*n* = 5 per group). Different superscripts (a–d) in the same column are significantly different at *p* < 0.05, using Duncan’s multiple range test. Normal, untreated group; Control, DNCB-sensitized group; Terfeadine, 250 μM Terfenadine-treated group; MP003-L, 125 μM MP003-treated group; and MP003-H, 250 μM MP003-treated group.

The expression of p-STAT3/STAT3, a key factor in AD, was measured in the skin tissues of DNCB-induced AD mice using Western blot analysis (Figure 6). Compared to the DNCB-induced control group, the expression of the phosphor-signal transducer and activator of transcription 3 (p-STAT3)/STAT3 was significantly (*p* < 0.05) reduced in the epidermal tissues of the MP003-treated group. These results suggest that MP003 effectively downregulates the activation of STAT3 in DNCB-induced AD skin lesions.

## 3. Discussion

In this study, we investigated the therapeutic potential of MP003, a peptide derived from hydrolyzed *Sebastes schlegelii* tail by-products, in ameliorating AD symptoms using a DNCB-induced NC/Nga mouse model. The results demonstrated that MP003 effectively reduced key symptoms of AD, including erythema, lichenification, collagen deposition, and skin thickness, highlighting its promise as a novel treatment for AD.

One of the most pronounced effects of MP003 is its ability to reduce erythema and lichenification, both of which are hallmarks of AD (Figure 2A). The efficacy of MP003 aligns with prior studies that have highlighted the anti-inflammatory properties of marine-derived bioactive peptides [27,28]. These peptides possess unique amino acid sequences that confer anti-inflammatory and antioxidative properties, reducing skin inflammation and promoting healing [29,30].

Excessive collagen deposition is a critical factor in the chronicity of AD lesions, often leading to skin fibrosis [31]. Our findings indicate that MP003 significantly decreases collagen fiber accumulation, mitigating the fibrotic changes often seen in chronic AD (Figure 2B). This effect is consistent with other studies on marine peptides, which have shown the ability to inhibit fibroblast proliferation and collagen synthesis [32]. This suggests that MP003 could be beneficial in not only reducing inflammation but also preventing long-term structural damage to the skin.

The reduction in epidermal and dermal thickness observed in MP003-treated mice may initially appear counterintuitive; however, it is a positive development for several reasons. In the context of AD, thickened skin (lichenification) is a sign of chronic inflammation and damage, often resulting from persistent scratching and irritation [33,34]. By reducing skin thickness, MP003 may help restore the skin’s natural barrier function, promote healing, and alleviate the symptoms associated with chronic AD [35,36]. This reduction indicates a shift away from the pathological thickening typically seen in chronic cases, suggesting that MP003 may assist in returning the skin to a healthier state [37].

The significant reduction in eosinophil and mast cell infiltration observed in MP003-treated mice indicates that MP003 plays a key role in modulating immune cell recruitment to inflamed skin tissues. Eosinophils and mast cells are well-known contributors to the inflammatory response in AD, exacerbating symptoms such as itching and tissue damage [35]. The ability of MP003 to reduce these immune cell populations suggests that it can modulate both acute inflammation and chronic immune dysregulation, which are characteristic of AD.

Elevated levels of cytokines such as IL-1β and IL-6, along with increased serum IgE, are indicative of the T helper 2 (Th2)-mediated immune response in AD [37,38]. MP003 treatment significantly lowers the serum levels of these cytokines and IgE, further supporting its role in downregulating the Th2 response. Prior studies on marine-derived peptides have similarly reported their capacity to suppress cytokine production, particularly those involved in inflammatory and allergic pathways [39]. This suggests that MP003’s mechanism of action may involve both the direct inhibition of pro-inflammatory cytokine production and the modulation of immune responses.

One of the novel findings of this study is the downregulation of p-STAT3/STAT3 protein expression in MP003-treated mice. STAT3 is a key transcription factor involved in AD pathogenesis, driving inflammation and contributing to skin barrier dysfunction [40,41]. By inhibiting STAT3 activation, MP003 appears to target one of the core molecular mechanisms driving chronic inflammation in AD, which is a promising finding for the development of targeted therapies.

Furthermore, the potential of using recycled fish-based by-products in the development of treatments for AD is an exciting avenue for future research. As this study demonstrates, MP003, derived from the tail by-products of *Sebastes schlegelii*, showcases the therapeutic benefits of utilizing marine resources. Recycled fish by-products are not only environmentally sustainable but also rich in bioactive compounds that could be harnessed for therapeutic purposes. The incorporation of these by-products into treatment formulations could offer new opportunities for addressing AD while promoting sustainability within the marine industry.

The findings from this study suggest that MP003 has significant potential as a therapeutic agent for AD. Marine peptides, due to their bioactive properties and unique amino acid profiles, are emerging as a valuable resource in the development of novel treatments for inflammatory diseases [42]. However, further research is necessary to elucidate the precise mechanisms by which MP003 modulates immune response and to assess its long-term efficacy and safety in clinical settings. Additionally, exploring the effects of MP003 on other skin conditions characterized by chronic inflammation and fibrosis could provide new insights into its broader therapeutic potential.

## 4. Materials and Methods

### 4.1. Materials

The reagents used in this study included 2,2′-Azinobis-(3-Ethylbenzthiazolin-6-Sulfonic Acid) (ABTS), potassium persulfate, dimethyl sulfoxide (DMSO), acetone, Tris, NaCl, sodium dodecyl sulfate, sodium deoxycholate, Triton X-100, ethylene-diamine-tetraacetic acid (EDTA), Phenylmethylsulfonyl fluoride (PMSF), 2,4-Dinitrochlorobenzene (DNCB), and Terfenadine, obtained from Sigma-Aldrich Chemical Co. (St. Louis, MO, USA). Phosphate-buffered saline (PBS) was sourced from Biosesang (Yongin-si, Gyeonggi-do, Republic of Korea). The hydrolytic enzymes Alcalase, Flavourzyme, Neutrase, and Protamex were provided by Novozyme (Bagsvaerd, Denmark). A 3500 Da dialysis membrane was acquired from Spectrum Labs (Gardena, CA, USA), and Acetonitrile was obtained from J.T. Baker (Phillipsburg, NJ, USA). The proteinase inhibitor cocktail and phosphatase inhibitor cocktail were obtained from Roche (Basel, Switzerland).

### 4.2. Production of Hydrolysate from Sebastes schlegelii Tail By-Products

This study utilized the tail by-products of *Sebastes schlegelii* collected from the fish market in Cheonan, Republic of Korea. The collected tails were freeze-dried (Vision, Daejeon, Republic of Korea) and ground into powder. For hydrolysis, 1 g of powdered fish tail by-products was mixed with 50 mL of distilled water (pH 7.0), 20 mg of hydrolytic enzyme, and 40 mM of Na_2_SO_3_ and incubated in a shaking incubator at 50 °C for 8 h. The reaction was terminated by heating at 100 °C. The hydrolysate was filtered using Whatman No. 41 filter paper (Whatman Ltd, Maidstone, Kent, UK) and then freeze-dried for storage.

### 4.3. Measurement of ABTS Radical Scavenging Activity

The ABTS radical scavenging activity of the hydrolysate was measured using the ABTS+· cation decolorization assay, as described by Re et al. [43]. A solution of the ABTS radical was generated by mixing 7 mM ABTS with 2.45 mM potassium persulfate and allowing it to react in the dark at room temperature (RT) for 24 h. The resulting ABTS radical solution was diluted with PBS (pH 7.4) to achieve an absorbance of 0.70 (±0.02) at 732 nm. The hydrolysate was diluted with PBS, and 20 μL of each dilution was mixed with 180 μL of the ABTS radical solution. After a 1 min reaction period, absorbance was measured at 732 nm.

### 4.4. Peptide Separation from Hydrolysate

To separate peptides from the hydrolysate, a 3500 Da dialysis membrane was initially used. The hydrolysate was dialyzed and then freeze-dried for the ABTS radical scavenging activity measurement. The high-activity fraction was further purified using size-exclusion chromatography (SuperdexTM 30 Increase 10/300 GL) at a flow rate of 0.5 mL/min. The resulting fractions were freeze-dried, and those with a high ABTS activity were analyzed using HPLC with a C18 column (ZORBAX SB-C18, 4.6 × 250 mm, 5 µm) at a flow rate of 1 mL/min. The most active fraction (STPO-B-II) was further purified using GPC (YMC-Pack Diol-60, 4.6 × 300 mm, 5 µm).

### 4.5. Amino Acid Sequence Analysis and Synthesis

The amino acid sequence of the isolated peptides was determined using an automated Edman degradation method with a protein sequencer (PPSQ-51A, Shimadzu, Kyoto, Japan).

### 4.6. Histological Evaluation in the DNCB-Induced AD Model

On the day of sacrifice, skin samples were collected from all groups and fixed in 10% formalin, then embedded in paraffin. Sections (4 μm thick) were stained with Masson’s trichrome, hematoxylin, and eosin (H&E), and toluidine blue to assess changes in epidermal and dermal thickness, collagen fiber density, and inflammatory cell infiltration [44].

### 4.7. Measurement of Cytokine and IgE Levels in Serum

Serum was obtained by centrifuging blood at 13,000× *g* at 4 °C for 15 min. The levels of IL-1β, IL-6, and IgE were quantified using mouse ELISA kits according to the manufacturer’s instructions. The Ig-E, IL-1β, and IL-6 ELISA kits were purchased from R&D Systems (Minneapolis, MN, USA).

### 4.8. RNA Extraction and RT-PCR

Inflammation-related genes *IL-1β* and *IL-6* were analyzed via RT-PCR in skin tissue from the atopic dermatitis model. RNA was extracted using TRIzol, and cDNA was synthesized from 1 μg of RNA using an RT premix (Bioneer, Daejeon, Republic of Korea). PCR amplification was performed using a PCR premix (Bioneer, Republic of Korea), and products were confirmed via electrophoresis on a 2% agarose gel. Primer sequences are detailed in Table 2. PCR was performed under the following conditions: initial denaturation at 94 °C for 5 min, followed by 40 cycles of denaturation at 94 °C for 30 s, annealing at 56 °C for 60 s, and extension at 72 °C for 60 s.

### 4.9. Western Blot Analysis

Western blotting was performed to assess the phosphorylation of STAT3 in the AD model. Skin samples were lysed in a buffer containing Tris-Cl (pH 7.4), NaCl, sodium dodecyl sulfate, sodium deoxycholate, Triton X-100, EDTA, a proteinase inhibitor, and a phosphatase inhibitor. Protein concentrations were determined using the BCA method. Equal amounts of protein were separated via sodium dodecyl-sulfate–polyacrylamide gel electrophoresis (SDS-PAGE) and transferred to a nitrocellulose membrane. Membranes were blocked with 5% skimmed milk in TBS-T and probed with primary and secondary antibodies. Protein expression was analyzed using the Enhanced Chemiluminescence kit (Thermo Scientific, Waltham, MA, USA). The antibodies used included anti-STAT3, anti-phospho-STAT3, anti-β-actin, and HRP-linked anti-rabbit IgG (Cell Signaling Technology, Beverly, MA, USA).

### 4.10. Statistical Analysis

Data are presented as the mean ± standard deviation (SD). Statistical significance was assessed using ANOVA, with *p* < 0.05 considered statistically significant.

## 5. Conclusions

This study demonstrates how peptides derived from hydrolyzed *Sebastes schlegelii* tail by-products, specifically MP003, exhibit potent antioxidative and anti-atopic dermatitis activities. MP003 significantly ameliorated the clinical symptoms of AD, including reductions in erythema, lichenification, collagen accumulation, and skin thickness, in a DNCB-induced AD mouse model. The peptide also suppressed immune cell infiltration and pro-inflammatory cytokine expression, highlighting its potential to modulate key inflammatory pathways associated with AD pathogenesis. These findings support the potential use of marine-derived bioactive peptides as novel therapeutic agents for inflammatory skin diseases such as AD. Further research is warranted to explore the underlying mechanisms and evaluate the safety and efficacy of MP003 in clinical settings. 

## Figures and Tables

**Table 1:** The yield and ABTS radical scavenging activity of *Sebastes schlegelii* tail by-products with 4 different proteases.

Hydrolysates	Yields of Protein Hydrolysates (%)	RC_50_ (μg/mL) ^1^
Alcalase	64.41	79.64 ± 2.64 ^b2^
Flavourzyme	75.20	77.23 ± 2.17 ^b^
Neutrase	73.36	97.70 ± 4.46 ^a^
Protamex	72.09	69.69 ± 0.41 ^c^

^1^ The concentration required for 50% reduction in ABTS radical at 1 min after starting the reaction. ^2^ Each value is the mean ± SD (*n* ≥ 3). Different superscripts (a–c) in the same column are significantly different at *p* < 0.05, using Duncan’s multiple range test.

**Table 2:** Primers used in this study.

Gene	Sequences	
*IL-1β*	Forward	5′-CATATGAGCTGAAAGCTCTCCA-3′
	Reverse	5′-GACACAGATTCCATGGTGAAGTC-3′
*IL-6*	Forward	5′-GGAGGCTTAATTACACATGTT-3″
	Reverse	5′-TGATTTCAAAGATGAATTGGAT-3′
*GAPDH*	Forward	5′-CCAGTATGACTCCACTCACG-3′
	Reverse	5′-CCTTCCACAATGCCAAGTT-3′

## Data Availability

The data are contained within the article and Supplementary Materials.

## Supplementary Materials

The following supporting information can be downloaded at https://www.mdpi.com/article/10.3390/md22100479/s1. Figure S1: Original photographs for the blots of each protein marker of Figure 6A.

## References

[1] Langan S.M., Irvine A.D., Weidinger S. (2020). Atopic Dermatitis. Lancet.

[2] Sroka-Tomaszewska J., Trzeciak M. (2021). Molecular Mechanisms of Atopic Dermatitis Pathogenesis. Int. J. Mol. Sci..

[3] Edslev S.M., Agner T., Andersen P.S. (2020). Skin Microbiome in Atopic Dermatitis. Acta Derm. Venereol..

[4] Fishbein A.B., Silverberg J.I., Wilson E.J., Ong P.Y. (2020). Update on Atopic Dermatitis: Diagnosis, Severity Assessment, and Treatment Selection. J. Allergy Clin. Immunol. Pract..

[5] Silverberg J.I. (2019). Comorbidities and the Impact of Atopic Dermatitis. Ann. Allergy Asthma Immunol..

[6] Puar N., Chovatiya R., Paller A.S. (2021). New Treatments in Atopic Dermatitis. Ann. Allergy Asthma Immunol..

[7] Food and Agriculture Organization of the United Nations [FAO] (2020). The State of World Fisheries and Aquaculture 2020: Sustainability in Action.

[8] Al Khawli F., Martí-Quijal F.J., Ferrer E., Ruiz M.J., Berrada H., Gavahian M., Barba F.J. (2020). Aquaculture and Its By-products as a Source of Nutrients and Bioactive Compounds. Adv. Food Nutr. Res..

[9] Zeller D., Cashion T., Palomares M., Pauly D. (2018). Global Marine Fisheries Discards: A Synthesis of Reconstructed Data. Fish Fish..

[10] Martí-Quijal F.J., Remize F., Meca G., Ferrer E., Ruiz M.J., Barba F.J. (2020). Fermentation in Fish and By-products Processing: An Overview of Current Research and Future Prospects. Curr. Opin. Food Sci..

[11] Ucak I., Afreen M., Montesano D., Carrillo C., Tomasevic I., Simal-Gandara J., Barba F.J. (2021). Functional and Bioactive Properties of Peptides Derived from Marine Side Streams. Mar. Drugs.

[12] Abhari K., Khaneghah A.M. (2020). Alternative Extraction Techniques to Obtain, Isolate and Purify Proteins and Bioactives from Aquaculture and By-Products. Adv. Food Nutr. Res..

[13] Dave D., Liu Y., Clark L., Dave N., Trenholm S., Westcott J. (2019). Availability of Marine Collagen from Newfoundland Fisheries and Aquaculture Waste Resources. Bioresour. Technol. Rep..

[14] León-López A., Morales-Peñaloza A., Martínez-Juárez V.M., Vargas-Torres A., Zeugolis D.I., Aguirre-Álvarez G. (2019). Hydrolyzed Collagen-Sources and Applications. Molecules.

[15] Sanchez A., Blanco M., Correa B., Perez-Martin R.I., Sotelo C.G. (2018). Effect of Fish Collagen Hydrolysates on Type I Collagen mRNA Levels of Human Dermal Fibroblast Culture. Mar. Drugs.

[16] Nasrollahi S.A., Ayatollahi A., Yazdanparast T., Samadi A., Hosseini H., Shamsipour M., Akhlaghi A.A., Yadangi S., Abels C., Firooz A. (2018). Comparison of Linoleic Acid-Containing Water-in-Oil Emulsion with Urea-Containing Water-in-Oil Emulsion in the Treatment of Atopic Dermatitis: A Randomized Clinical Trial. Clin. Cosmet. Investig. Dermatol..

[17] Aguirre-Cruz G., León-López A., Cruz-Gómez V., Jiménez-Alvarado R., Aguirre-Álvarez G. (2020). Collagen Hydrolysates for Skin Protection: Oral Administration and Topical Formulation. Antioxidants.

[18] Henriques A., Vázquez J.A., Valcarcel J., Mendes R., Bandarra N.M., Pires C. (2021). Characterization of Protein Hydrolysates from Fish Discards and By-Products from the North-West Spain Fishing Fleet as Potential Sources of Bioactive Peptides. Mar. Drugs.

[19] Atef M., Chait Y.A., Ojagh S.M., Latifi A.M., Esmaeili M., Hammami R., Udenigwe C.C. (2021). Anti-Salmonella Activity and Peptidomic Profiling of Peptide Fractions Produced from Sturgeon Fish Skin Collagen (*Huso huso*) Using Commercial Enzymes. Nutrients.

[20] Kim H.Y., Lee G.O., Shin J., Kim C.E., Kang G.H., Kim S.H., Kang H.W., Lee H.S., Kim J.Y. (2022). Validation of an Analytical Method for the Quantification of a Marker Compound and Determination of Its Biological Activities in Skate Skin Collagen Peptides. Korean J. Food Preserv..

[21] Pihlanto-Leppala A. (2000). Bioactive Peptides Derived from Bovine Whey Proteins: Opioid and ACE-Inhibitory Peptides. Trends Food Sci. Technol..

[22] Atef M., Mahdi Ojagh S. (2017). Health Benefits and Food Applications of Bioactive Compounds from Fish By-products: A Review. J. Funct. Foods.

[23] Jo C., Khan F.F., Khan M.I., Iqbal J. (2017). Marine Bioactive Peptides: Types, Structures, and Physiological Functions. Food Rev. Int..

[24] Wang X., Yu H., Xing R., Li P. (2017). Characterization, Preparation, and Purification of Marine Bioactive Peptides. BioMed. Res. Int..

[25] Luo Z., Dong J., Zhang Z., Xu X., Zhang X. (2021). Microsatellite-Based Parentage Analysis of Offspring Conducted in Different Regions of the Black Rockfish (*Sebastes schlegelii*) Ovary. J. Fish. Sci. China.

[26] Gao T., Han Z., Zhang X., Luo J., Yanagimoto T., Zhang H. (2016). Population Genetic Differentiation of the Black Rockfish (*Sebastes schlegelii*) Revealed by Microsatellites. Biochem. Syst. Ecol..

[27] Wijesekara I., Kim S.K. (2010). Angiotensin-I-Converting Enzyme (ACE) Inhibitors from Marine Resources: Prospects in the Pharmaceutical Industry. Mar. Drugs.

[28] Lee S.G., Hwang J.W., Kang H. (2024). Antioxidant and Skin-Whitening Efficacy of a Novel Decapeptide (DP, KGYSSYICDK) Derived from Fish By-Products. Mar. Drugs.

[29] Kim S.K., Wijesekara I. (2010). Development and Biological Activities of Marine-Derived Bioactive Peptides: A Review. J. Funct. Foods.

[30] Hwang J.W., Lee S.G., Kang H. (2024). Antioxidant, Antibacterial Properties of Novel Peptide CP by Enzymatic Hydrolysis of *Chromis notata* By-Products and Its Efficacy on Atopic Dermatitis. Mar. Drugs.

[31] Irvine A.D., McLean W.H., Leung D.Y. (2011). Filaggrin Mutations Associated with Skin and Allergic Diseases. N. Engl. J. Med..

[32] Kim Y.M., Park Y.S., Yoon T.S., Lee J.S., Kim J.S. (2019). Marine Peptides Attenuate Fibrotic and Inflammatory Markers In Vitro. Int. J. Mol. Sci..

[33] Paller A.S., Eichenfield L.F., Tom W.L. (2019). Atopic Dermatitis: A Practical Guide for Clinicians. J. Allergy Clin. Immunol..

[34] Wollenberg A., Schmidt H., Köneke J. (2018). Atopic Dermatitis: From Pathophysiology to Treatment. Ther. Adv. Chronic Dis..

[35] Rawlings A.V., Harding C.R. (2004). Moisturization and Skin Barrier Function. Dermatol. Ther..

[36] Darlenski R., Tsankov N. (2010). Skin Barrier Function. Dermatology.

[37] Gittler J.K., Shemer A., Suárez-Fariñas M., Fuentes-Duculan J., Gulewicz K.J., Wang C.Q., Mitsui H., Cardinale I., de Guzman Strong C., Krueger J.G. (2012). Progressive Activation of Th2/Th22 Cytokines and Selective Epidermal Proteins Characterizes Acute and Chronic Atopic Dermatitis. J. Allergy Clin. Immunol..

[38] Udenigwe C.C., Aluko R.E. (2012). Food Protein-Derived Bioactive Peptides: Production, Processing, and Potential Health Benefits. J. Food Sci..

[39] Su Y., Li X., Ma W., Yuan L., Wang K., Xiao R. (2020). The Effects of Marine-Derived Bioactive Peptides on the Immune Response and Gut Microbiota. Mar. Drugs.

[40] Sano S. (2013). Role of STAT3 in Inflammatory Dermatoses. JAK-STAT.

[41] Levy D.E., Lee C.K. (2002). What Does Stat3 Do?. J. Clin. Investig..

[42] Fan Z., Zhao L., Wang Z., Zheng X., Gong M. (2020). Marine Peptides as Potential Agents for the Prevention and Treatment of Chronic Inflammatory Diseases. J. Funct. Foods.

[43] Re R., Pellegrini N., Proteggente A., Pannala A., Yang M., Rice-Evans C. (1999). Antioxidant Activity Applying an Improved ABTS Radical Cation Decolorization Assay. Free Radic. Biol. Med..

[44] Zhang Q., Wang H., Ran C., Lyu Y., Li F., Yao Y., Xing S., Wang L., Chen S. (2023). Anti-Inflammatory Effects of Amarogentin on 2,4-Dinitrochlorobenzene-Induced Atopic Dermatitis-like Mice and in HaCaT Cells. Anim. Model. Exp. Med..

