# Supplementary material for: Antioxidative and Anti-Atopic Dermatitis Effects of Peptides Derived from Hydrolyzed Sebastes schlegelii Tail By-Products
Source: Mar Drugs. 2024 Oct 19;22(10):479. doi: 10.3390/md22100479 (PMC11509535; doi:10.3390/md22100479)
Supplement: Supplementary file 1 [file marinedrugs-22-00479-s001.zip › marinedrugs-3244182-supplementary.pdf]

Original Images for Blots/Gels

Supplementary Figure S1. Original photographs for the blots of each protein marker of Figure 6A.

Figure 6

(A)

MP003 (µM)	0	0	0	125	250
Terfenadine (µM)	0	0	250	0	0
DNCB	-	+	+	+	+

p-STAT3

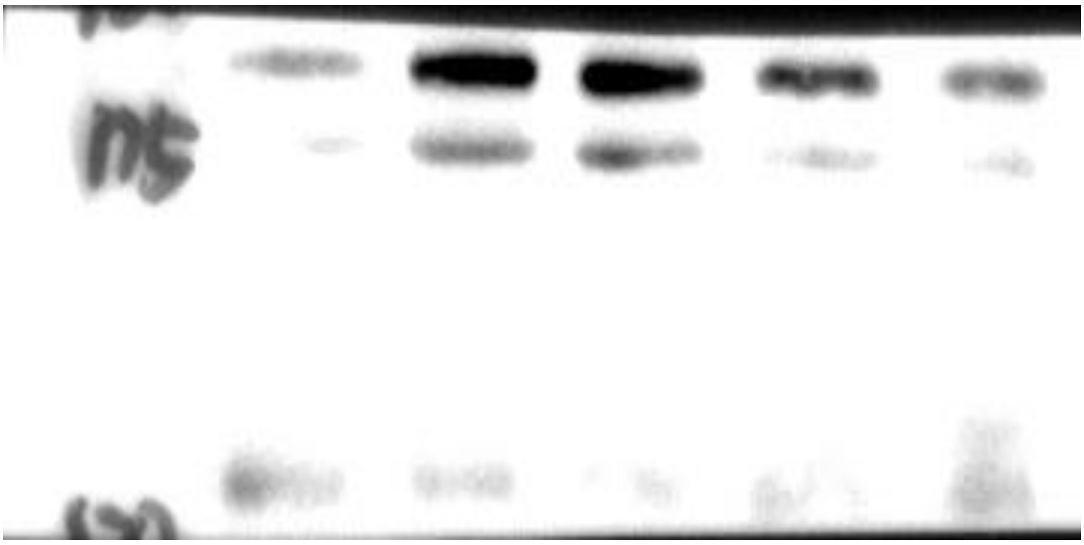

86 kDa

MP003 (µM)	0	0	0	125	250
Terfenadine (µM)	0	0	250	0	0
DNCB	-	+	+	+	+

STAT3

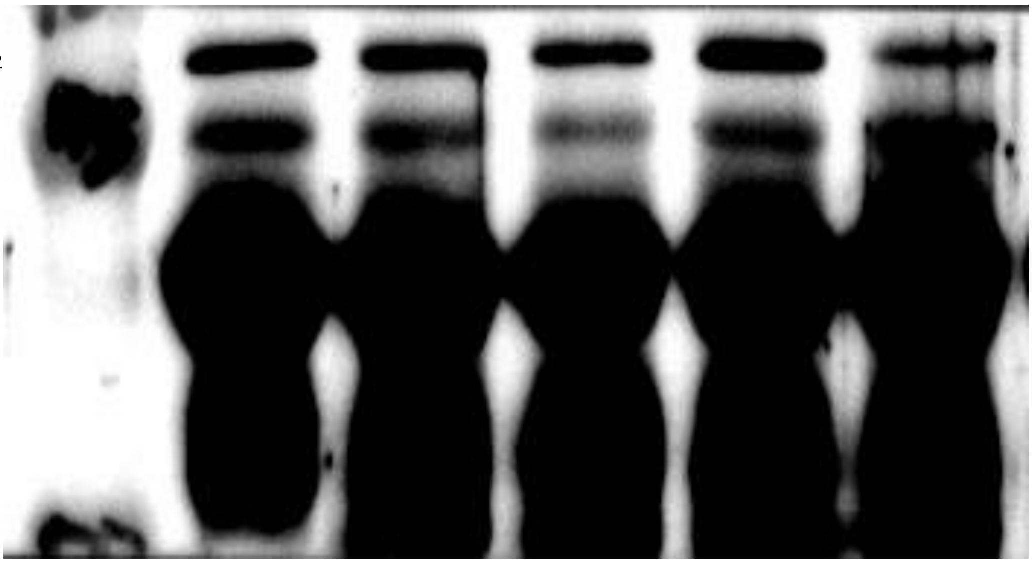

86 kDa

MP003 (µM)	0	0	0	125	250
Terfenadine (µM)	0	0	250	0	0
DNCB	-	+	+	+	+

β-actin

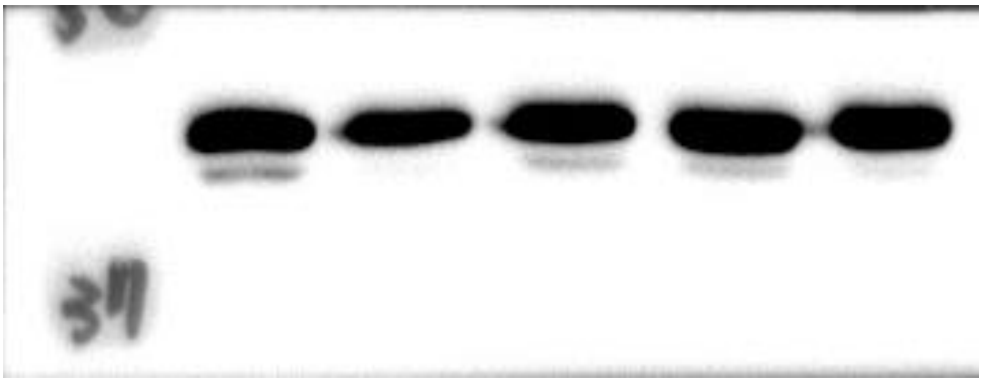

45 kDa
